# G protein-coupled kisspeptin receptor induces metabolic reprograming and tumorigenesis in estrogen receptor-negative breast cancer

**DOI:** 10.1038/s41419-020-2305-7

**Published:** 2020-02-07

**Authors:** Magdalena Dragan, Mai-Uyen Nguyen, Stephania Guzman, Cameron Goertzen, Muriel Brackstone, Waljit S. Dhillo, Paul R. Bech, Sophie Clarke, Ali Abbara, Alan B. Tuck, David A. Hess, Sharon R. Pine, Wei-Xing Zong, Frederic E. Wondisford, Xiaoyang Su, Andy V. Babwah, Moshmi Bhattacharya

**Affiliations:** 1 Department of Medicine, Robert Wood Johnson Medical School, Rutgers University, New Brunswick, NJ USA; 2 Cancer Invasion and Metastasis Laboratory, Faculty of Dentistry, University of Toronto, Toronto, ON Canada; 3 Department of Surgery, London Health Sciences Centre, London, ON Canada; 4 Section of Investigative Medicine, Imperial College London, London, UK; 5 Department of Pathology, The University of Western Ontario, London, ON Canada; 6 Department of Physiology and Pharmacology, The University of Western Ontario, London, ON Canada; 7 Rutgers Cancer Institute of New Jersey, Rutgers University, New Brunswick, NJ USA; 8 Department of Chemical Biology, Ernest Mario School of Pharmacy, Rutgers University, New Brunswick, NJ USA; 9 Child Health Institute of New Jersey, New Brunswick, NJ USA; 10 Department of Pediatrics, Robert Wood Johnson Medical School, Rutgers University, New Brunswick, NJ USA

**Keywords:** Breast cancer, Cancer metabolism, Metastasis

## Abstract

Triple-negative breast cancer (TNBC) is a highly metastatic and deadly disease. TNBC tumors lack estrogen receptor (ERα), progesterone receptor (PR), and HER2 (ErbB2) and exhibit increased glutamine metabolism, a requirement for tumor growth. The G protein-coupled kisspeptin receptor (KISS1R) is highly expressed in patient TNBC tumors and promotes malignant transformation of breast epithelial cells. This study found that TNBC patients displayed elevated plasma kisspeptin levels compared with healthy subjects. It also provides the first evidence that in addition to promoting tumor growth and metastasis in vivo, KISS1R-induced glutamine dependence of tumors. In addition, tracer-based metabolomics analyses revealed that KISS1R promoted glutaminolysis and nucleotide biosynthesis by increasing c-Myc and glutaminase levels, key regulators of glutamine metabolism. Overall, this study establishes KISS1R as a novel regulator of TNBC metabolism and metastasis, suggesting that targeting KISS1R could have therapeutic potential in the treatment of TNBC.

## Introduction

Triple-negative breast cancer (TNBC) is a particularly deadly subtype of breast cancer. TNBC patient survival is limited by extensive metastasis and the frequent development of tumor resistance to available chemotherapy^1^. These patients fail to respond to endocrine or anti-HER2 therapy and lack targeted therapies^1^. Clearly, in order to identify clinically relevant biomarkers and develop targeted therapies, there is a vital need to better understand molecular pathways that are distinctly activated in TNBC.

Metabolic reprogramming is a hallmark of cellular transformation; this enables cancer cells to maintain high proliferation rates despite fluctuations in nutrient availability^2^. In highly proliferative cells, glutamine can provide energy via the tricarboxylic acid (TCA) cycle, as well as nitrogen for nucleotide biosynthesis. Glutamine is converted to glutamate by glutaminase, the rate-limiting enzyme in glutaminolysis^3^. Aggressive basal-like tumors such as TNBC are dependent on exogenous glutamine for survival and growth^4^ and high glutamate levels have been found in human TNBC tumors^5–7^.

G protein-coupled receptors (GPCRs), targets for over a third of all marketed pharmaceuticals, regulate tumorigenesis^8^, but little is known about their roles in regulating tumor metabolism. The GPCR, kisspeptin 1 receptor (KISS1R previously known as GPR54) binds kisspeptins (KPs), a family of secreted, biologically active peptides found in the blood^9,10^. KPs (10, 13, 14, or 54 amino acids) are derived from a 145 amino acid peptide, KISS1 (encoded by the *KISS1* gene) that is cleaved rapidly in the serum by matrix metalloproteinases (MMPs) MT1-MMP, MMP-9, and furin^11,12^. All KPs have similar affinity for KISS1R; however, KP-10 is the agonist of choice for most studies^13–15^. *KISS1* is commonly classified as a metastasis suppressor gene and exerts antitumorigenic roles in many cancers^10,16,17^. However, when breast cells lose ERα, KISS1R signaling promotes epithelial-to-mesenchymal-transition (EMT) and stimulates tumor invasion by inducing invadopodia formation via MT1-MMP and the mitogen-activated protein kinase (MAPK) pathway^18,19^. KISS1R activation also induces TNBC invasion by activating the epidermal growth factor receptor, via MMP-9, but fails to activate HER2^16–20^.

Mechanistically, we and others have shown that ERα negatively regulates *KISS1*^21^ and *KISS1R* levels, as well as KISS1R-induced invasion^17–20^. Therefore, in ERα-negative cancers such as TNBC, this may partly account for the switching of KISS1R from metastasis suppressor to promoter. This dualistic nature of signaling molecules where they exhibit pro- and antitumor roles is not unique to *KISS1*/*KISS1R*. For example, c-Myc^22^, AMP-activated protein kinase^23^, and transforming growth factor β^24^ are among the many reported to play dual roles, highlighting the importance of studying cancer in context.

Recently, we reported that KISS1/KISS1R expression are upregulated in TNBC patient tumor biopsies compared with healthy breast tissue and that KISS1R signaling induces a drug-resistant phenotype in TNBC^25^. Human TNBC cells express high levels of both KISS1 and KISS1R compared with normal mammary epithelial cells and secrete KP-10^20,25^. However, it is not known whether plasma levels of KPs change in TNBC and whether KISS1R regulates metastasis or tumor metabolism.

This study reveals that KISS1R signaling plays a central and multifunctional role in ERα-negative breast cancer, by supporting tumor growth and metastasis. It also uncovers that KISS1R signaling metabolically reprograms cancer cells to depend on glutamine for tumorigenesis. Specifically, KISS1R regulates the expression of the transcription factor c-Myc and glutaminase that are necessary for glutamine catabolism and therefore drives processes such as nucleotide synthesis required for tumor growth.

## Methods

### Blood collection and plasma kisspeptin measurements

The study was approved by the Office of Human Research Ethics, Western University and all female participants provided informed consent. Blood (5 mL) was collected in BD Vacutainer K2 EDTA tubes (VWR International) from the following groups: healthy subjects (*n* = 26), nonmetastatic “early” TNBC patients (*n* = 26), metastatic TNBC patients (*n* = 27) presenting to Dr. M Brackstone’s Breast Care Clinic at St. Joseph’s Health Care London or at the London Regional Cancer Program (see Table 1 for patient demographics). Blood was centrifuged at 3000 rpm for 10 min and the plasma was collected and frozen immediately in liquid nitrogen. Samples were stored at −80 °C and subsequently thawed to quantify plasma KP using a sensitive in-house radioimmunoassay, in the Dhillo laboratory as described^26^. Statistical analysis of clinical samples was conducted by a biostatistician (Statistical Services, Western University) using a nonparametric Kruskal–Wallis test.

**Table 1:** Clinical profile of study participants (females: healthy subjects and TNBC patients) from London Health Science Center, London, Ontario, Canada.

Characteristics	Number (range)	KP (pmol/L) (mean ± SEM)
Normal subjects age (years) (*n* = 26)	Mean 34 (20–52)	39.1 ± 2.3
Early disease (nonmetastatic) TNBC age (years) (*n* = 25)	Mean 54.8 (27–79)	78 ± 6.9
Tumor size (mm)	Mean 25.3 (4–55)	
Blood collection		
Before any treatment	18	77.83 ± 5.5
Before any surgery	21	82.55 ± 6.1
During/after chemotherapy	3	96.6 ± 7.1
After tumor removing surgery	4	68.3 ± 6.7
After radiation therapy	0	
Tumor size		
T1	13	
T2	7	
T3	1	
T4	4	
Node status		
N0	16	
N1	5	
N2	2	
N3	1	
Nx	1	
Metastatic disease TNBC age (years) (*n* = 27)	Mean 62.1 (40–85)	64.9 ± 6.8
No surgery (primary tumor present)	8	66.0 ± 5.5
Surgery (tumor removed)	19	64.4 ± 6.1
No chemotherapy	12	77.8 ± 5.4
Chemotherapy	13	62.1 ± 7.1
Radiation therapy	12	73.9 ± 6.6
Site of metastasis		
Brain	5	
Lung/pleura	12	
Bone	7	
Lymph nodes/lymphatics	10	

#### Immunohistochemistry

Paraffin-embedded mammary tissue from nondiseased and breast cancer patients (TNBC) were obtained from London Health Science Center University Hospital, London, Ontario, Canada following approval by the University of Western Ontario Research Ethics Board. Immunohistochemical analysis of human breast tissue was done as previously described^27^. In short, following deparaffinization and heat induced antigen retrieval, slides were incubated with the following antibodies: rabbit monoclonal antikeratin 5 CST 71536 (1:1000) and mouse monoclonal antikeratin 8/18 CST 4546 (1:100) from Cell Signaling Technology; rabbit monoclonal anti-c-Myc (1:500) ab32072 and rabbit monoclonal anti-glutaminase ab156876 (1:100) from Abcam.

#### Cell culture

Human cell lines were purchased from ATCC and maintained at 37 °C with 5% CO_2_. ERα**-**negative human breast cancer SKBR3 cells and TNBC MDA-MB-231 were cultured in RPMI 1640 supplemented with 10% (v/v) fetal bovine serum (FBS). Stable knockdown of KISS1R in MDA-MB-231 cells has been previously described^19^. Expression of shRNA did not affect cell viability^19^. SKBR3 cell lines stably expressing KISS1R (SKBR3FLAG-KISS1R) and pFLAG vector controls were generated, as described^18^ and were grown in media containing G418 (1 μg/mL). KISS1R expression was verified weekly by western blot. All stable cells lines represent polyclonal (mixed) cell populations.

#### c-MYC depletion by siRNA

Cells were transfected with siMYC or control siRNA (Silencer™ Select Pre-Designed siRNA, Thermo) according to manufacturer instructions. c-Myc expression was determined by RT-qPCR and western blot analysis 48 h and 72 h post transfection.

#### Immunoblot assays

Immunoblot assays were conducted as previously described^18,20^. For human breast tissue and patient TNBC tumor protein analysis, immunoblots were conducted using TNBC biopsies obtained from Dr. Brackstone’s London Tumor Biobank, in accordance with the Health Sciences Research Ethics Board at the University of Western Ontario, London, Ontario, Canada. Core tumor tissues (10 mm × 1 mm) were collected by guided needle biopsy and immediately frozen in liquid nitrogen and the diagnosis was confirmed by the pathologist. Normal breast tissue (noncancerous) was also obtained from the London Tumor Biobank. The cores were homogenized in RIPA lysis buffer containing proteases inhibitors and centrifuged at 4 °C and protein expression in 100 μg lysates was analyzed by western blot analysis. The relative expression of each protein to GADPH was also normalized to their expression in MDA-MB-231 cell lysates (20 μg) that served as an internal control in all studies.

For cultured cells, cell lysates were prepared following incubation in RIPA buffer for 20 min at 4 °C. Protein (50 μg) was separated using SDS-PAGE and probed using the following antibodies: Abcam (rabbit anti-KISS1R (ab137483), rabbit anti-NAGS (ab65536), both 1:1000), Cell Signaling Technology (rabbit anti-c-Myc (CST #13987), mouse antikeratin 8/18 CST 4546, all 1:1000), rabbit anti-glutaminase (1:500, Invitrogen 710997), rabbit anti-glutamine synthetase (1:5000, Sigma-Aldrich G2781), rabbit anti-Erk (1:1000), and manti-Phospho-Erk (1:2000) from New England Biolabs. Mouse anti-GAPDH (1:4000, GeneTex GTX627408), anti-β actin (1:5000, Thermo Fisher Scientific APPA6889) or anti-vinculin (1:1000, Bio-Rad MCA465GA) were used for loading control. Protein was then incubated for 1 h in horseradish peroxidase (HRP)-conjugated rabbit (1:2500, Cell Signaling Technology, CST7074S) or mouse (1:2500, GE Healthcare, NA931) secondary antibody. For ERK activation assays, cells were serum starved for 3 h prior to 24 h treatment with 10 μM of U0126 (active MEK1 and MEK2 inhibitor) or U0124 (inactive U0126 analog serving as negative control). Expression levels were imaged by chemiluminescence with SuperSignal West Dura Extended Duration Substrate (Thermo Scientific) and ChemiDoc Touch imaging system (Bio-Rad) and subsequently quantified using Image Lab Software (Bio-Rad).

#### Xenograft assays

Animal studies were performed in accordance with the Institutional Animal Care and Use Committee (IACUC) guidelines of Rutgers University and under a protocol approved by the Western University Animal Care Committee with the recommendations of the Canadian Council on Animal Care. Six-week-old female NOD/SCIDIL2Rγ null (immunocompromised) mice were utilized for all assays and experiments were conducted as previously described^27^. Sample size for xenografts was estimated based on law of diminishing return by statistician. Mice were randomly assigned to control or experimental group, for each xenograft study (spontaneous metastasis assay or experimental metastasis assay) and the investigator was blinded to group allocation or when experimental outcome was assessed. For spontaneous metastasis assay, human metastatic TNBC MDA-MB-231 cells stably expressing scrambled control or two individual *KISS1R*-specific shRNA^19^ were resuspended in growth factor-reduced Matrigel (1:1) (1 × 10^6^ cells/mouse) and injected into the right mammary fat pad of mice. Subsequent tumor growth was measured by caliper biweekly. At 5 weeks post injection, mice were sacrificed, and primary tumor and lung tissues were harvested, fixed in 4% paraformaldehyde and embedded in paraffin. For orthotopic and experimental metastasis assays, we used a *gain-of-function* model using ERα-negative human SKBR3 breast cancer cells stably expressing FLAG-KISS1R or pFLAG control cell lines generated as described^18^. Cells were injected into the tail vein or mammary fat pads of 6-week-old immunocompromised mice for experimental metastasis or orthotopic xenograft models, respectively. Mice were sacrificed at 3 weeks for lung colonization and 6–8 weeks to assess primary tumor growth. Lungs and primary tumors were harvested, fixed in 4% paraformaldehyde, and processed for histology as described previously^27^. Sections were stained as previously described^27^ using antihuman Ki67 (1:100 dilution, Thermo Fisher Scientific), antihuman cytochrome C oxidase subunit II (1:100, Abcam) or rabbit anti-glutaminase (1:500, Thermo Fisher). Lung metastatic tumor burden and the number of metastases were quantified in antihuman cytochrome C oxidase and hematoxylin and eosin stained lung sections using Aperio ImageScope software; slides were reviewed by the pathologist (Dr. A. Tuck, London Health Sciences Center).

#### Quantitative real-time PCR (qPCR)

Total RNA was extracted from cells using the RNeasy Mini Kit (Qiagen) and reverse-transcription was carried out according to manufacturer’s instructions using iScript RT Supermix (Bio-Rad). Gene expression was determined using SYBR green real-time qPCR (RT-qPCR) as previously described^25^. The steady-state mRNA levels of each gene of interest was determined by amplification of cDNA using specific primers and the results were normalized to β-actin. Specific primers to determine the mRNA levels of each gene include: *GLS* (glutaminase) forward primer (F): AGCTTGTGTGGTCTTCCATGAT and reverse primer (R): TCATGAAGCTAGGGTGAGAGAGA; *GLUL* (F): GGACAATGCCCGACGTCTAA and (R): AGAAGACACGTGCGGATGAG; *CPSII* (F): AGACGCCTATGGCAACTGTG and (R): GTCTGCCTCAGGAGCTGATAC. *MYC, SCL1A5 and GLUD* primers were purchased from Bio-Rad (validated PCRPrime primers).

#### Cell growth assays

For glutamine deprivation assay, SKBR3FLAG-KISS1R cells and controls were seeded in 6 cm dishes (400,000 cells each) in glutamine-free RPMI media with dialyzed FBS. Cells were treated with 0.02 mM glutamine, 0.2 mM glutamine, or 2 mM glutamine (Gibco) over 72 h; media was changed every 24 h and cells trypsinized and counted using a hemocytometer at 24 h intervals. For BPTES or CB-839 (Sigma Aldrich) treatment, SKBR3FLAG-KISS1R cells (400,000 cells) were plated in 6 cm dishes. On the following day, these cells were treated with different concentrations of BPTES or CB-839 and cell number counted at 24 h intervals. To determine the effect of c-Myc knockdown on cell growth, SKBR3FLAG-KISS1R cells expressing c-Myc siRNA were cultured in media without glutamine. Media was changed daily and each day cells were counted for each experimental condition.

#### Scratch assays

These assays were conducted as described^25,27^. SKBR3FLAG-KISS1R cells expressing siMYC shRNA or scrambled controls were plated in duplicate wells, grown to confluence in a 12-well plate, and scratched with a sterile pipette tip. Cells in FBS supplemented media were allowed to migrate into the scratch for 18 h, as previously shown^25^. Images were taken using a microscope (EVOSTM FL Imaging System). For each image (per time point), the width of the scratch (μm) was measured at sevenpoints along the scratch. The distance migrated was calculated by subtracting the width of the scratch at each time point from the width of the scratch at time zero. The distances migrated into the scratch at each of the sevenpoints/image was averaged to determine the distance migrated for each well. Cell migration was expressed as fold over scrambled control. Cells were counted with trypan blue to determine cell viability.

#### Metabolomic analysis by liquid chromatography–mass spectrometry (LC–MS)

Metabolites from serum (10 μL samples) were extracted using 40 μL of ice-cold methanol, incubated for 20 min at −20 °C and then centrifuged for 10 min at 16,000 × *g* and 4 °C. The supernatant was subsequently transferred to clean tubes; pellets were extracted again with 200 μL of 40:40:20 methanol:acetonitrile:H2O and sat on ice for 10 min prior to centrifugation for 10 min at 16,000 *g* and 4 °C. Combined supernatants from the first and second extractions (~240 μL total) were further processed with Phospholipid Removal 1 mL tube (Phenomenex) according to the manufacturer’s instructions. Final extracts were stored at −80 °C until analysis using LC–MS.

For metabolite extraction from tumors, 25 mg samples were pulverized using a Cryomill (Retsch) in liquid nitrogen for 2 min at 25 Hz. Five hundred microliters of 40:40:20 methanol: acetonitrile: H_2_O with 0.5% formic acid solution at −20 °C was added to the ground samples before vortexing and centrifuging for 10 min at 16,000 × *g* and 4 °C. Supernatant was subsequently transferred to clean tubes; the previous step was repeated to extract the pellets again. Supernatants from the first and second extractions were combined and spun down for 10 min at 16,000 × *g* at 4 °C to remove protein precipitate from the supernatant. Five hundred microliters of extract was then transferred to a clean tube and neutralized with 44 μL of 15% NH4HCO3 solution, resulting in the final extract (stored at −80 °C until analysis using LC–MS).

For metabolite extraction from cultured cells, SKBR3FLAG-KISS1R and SKBR3pFLAG (control) cells were plated, left to adhere for 24 h and then serum starved in RPMI 1640 for 24 h. For experiments with labeled glutamine, RPMI media without glutamine was used, containing labeled l-Glutamine [U-^13^C] or [U-Amide-^15^N] (Cambridge Isotope Laboratories, Tewksbury, MA) at a 2 mM concentration. Conditioned media was collected at 4 and 16 h, spun to remove debris, and frozen until analysis. Cell metabolites were obtained by lying the cells in ice-cold lysis buffer consisting of 40:40:20 methanol:acetonitrile:water with 0.5% formic acid. After incubation on ice for 5 min, 15% NH_4_HCO_3_ was added to each plate. Lysates were then centrifuged at 15,000 × *g* for 10 min at 4 °C and the supernatant was transferred to new tubes and stored at −80 °C. All samples were diluted 50× in a 40:40:20 methanol:acetonitrile:water with 0.5% formic acid and loaded in LC–MS for analysis. Each condition was done in triplicate. Normalization of pool size was done to pack cell volume, as well as to protein amount per dish.

#### LC–MS analysis

LC–MS analysis of cell metabolites was conducted on Q Exactive Plus Hybrid Quadrupole-Orbitrap mass spectrometer (Thermo Fisher Scientific) alongside hydrophilic interaction chromatography. The Dionex UltiMate 3000 UHPLC system (Thermo Fisher Scientific) with XBridge BEH amide column (Waters, Milford, MA) and XP VanGuard Cartridge (Waters, Milford, MA) were used for LC separation. The LC gradient, comprised of solvent A (95%:5% H_2_O:acetonitrile with 20 mM ammonium acetate, 20 mM ammonium hydroxide, pH 9.4) and solvent B (20%:80% H_2_O:acetonitrile with 20 mM ammonium acetate, 20 mM ammonium hydroxide, pH 9.4), corresponded with the following solvent B percentages over time: 0 min, 100%: 3 min, 100%; 3.2 min, 90%; 6.2 min, 90%; 6.5 min, 80%; 10.5 min, 80%; 10.7 min, 70%; 13.5 min, 70%; 13.7 min, 45%; 16 min, 45%; 16.5 min, 100%. Chromatography flow rate was at 300 μL/min and injection volume 5 μL. Column temperature was maintained at 25 °C. MS scans were set to negative ion mode with a resolution of 70,000 at *m*/*z* 200, in addition to an automatic gain control target of 3 × 10^6^ and scan range of 75–1000. Metabolite data were obtained using the MAVEN software package as previously described^28^ with each labeled isotope (mass accuracy window: 5 ppm). Labeled isotope natural abundance and impurity were corrected using the AccuCor package coded in R, as previously described^29^.

## Results

### Plasma kisspeptin levels and glutamine metabolism in human TNBC

**Fig. 1:**
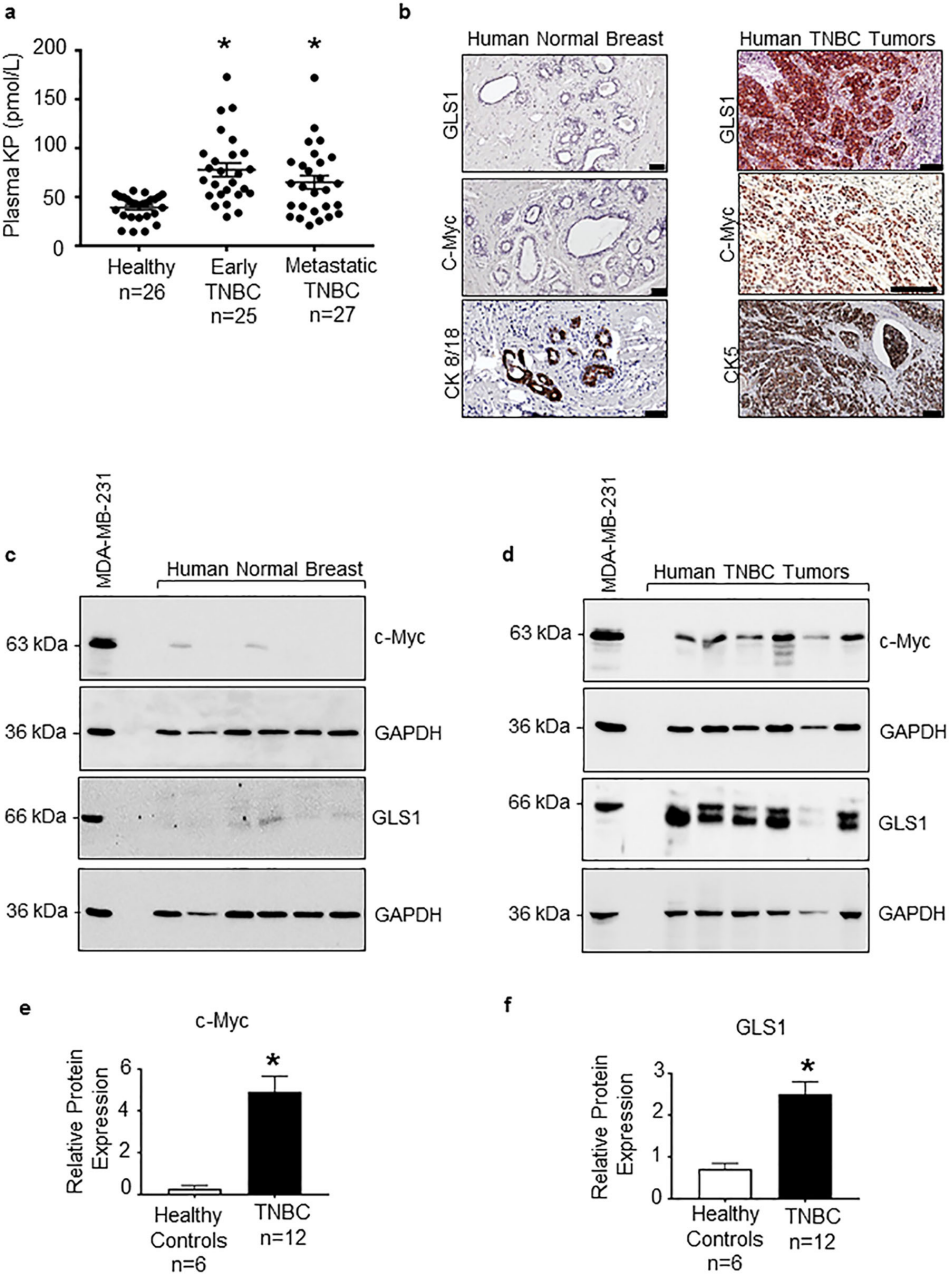
Plasma kisspeptin, c-Myc, and glutaminase are highly expressed in human triple-negative breast cancer (TNBC). **a** Plasma kisspeptin (KP) levels (pmol/L; mean ± SEM) measured by radioimmunoassay in blood samples taken from healthy subjects (*n* = 26), nonmetastatic TNBC patients (i.e., early disease; *n* = 25), or metastatic TNBC patients (*n* = 27). Statistical analysis done using a nonparametric Kruskal–Wallis test. Error bars: SEM. **b** Representative images of immunostaining on formalin-fixed and paraffin-embedded human breast tissue biopsies showing the lack of expression of GLS or c-Myc in normal breast tissue, whereas these proteins are enriched in TNBC tumors; staining of epithelial markers cytokeratins 8/18 (enriched in normal breast tissue mammary glands) and cytokeratin 5 (enriched in basal tumors) shown below. Scale bar, 50 μM; *n* = 5/cohort. Representative western blots showing the expression of endogenous c-Myc and glutaminase (GLS1) in breast tissue lysates from (**c**) six healthy subjects and (**d**) 12 TNBC primary tumor biopsies, relative to expression of each protein in MDA-MB-231 cell lysates (positive control); see Supplementary Fig. 1a for the remaining six TNBC blots. Densitometric analysis of **e** c-Myc and **f** GLS1 blots from 12 TNBC biopsies and six normal breast tissue, conducted by normalizing to GADPH loading controls and protein expression in MDA-MB-231 cell lysates (internal control); Student’s unpaired *t* test, *p < 0.05.

Human TNBC cells secrete KPs^25^, and *KISS1* and *KISS1R* mRNA are elevated in human TNBC patient tumors compared with healthy breast samples^25^. However, whether plasma KP levels differ in TNBC patients compared with healthy subjects remains unknown. Thus, plasma KP levels were measured among the following patient groups, as previously described^30^ (see Table 1 for patient demographics): (1) newly diagnosed, nonmetastatic TNBC (early disease); (2) metastatic TNBC (advanced disease), and (3) healthy subjects (no prior history of breast cancer). The data revealed that plasma KP levels in TNBC patients were significantly higher compared with the levels observed in healthy females (Fig. 1a, nonmetastatic TNBC: 78 ± 5.5 pmol/L (*p* < 0.0001), metastatic TNBC: 64.9 ± 6.6 pmol/L (*p* = 0.017), and healthy: 39.1 ± 2.3 pmol/L).

TNBC tumors display dysregulated glutamine metabolism^5,31^. Similar to previous reports^4,32,33^, c-Myc and glutaminase (encoded by GLS1) were found to be significantly upregulated in human primary TNBC tumor biopsies compared with healthy breast tissue (Fig. 1b–f; see Supplementary Fig. 1a for remaining blots); the expression of both isoforms of GLS1 (KGA, kidney-type glutaminase and GAC, glutaminase C) was observed in TNBC biopsies (Fig. 1d, Supplementary Fig. 1a). The presence of epithelial cells in breast tissue was identified using cytokeratins, markers for epithelial cells^34–36^. Specifically, mammary glands in normal breast tissue expressed cytokeratins 8/18 (Fig. 1b, Supplementary Fig. 1b), whereas basal TNBC tumors robustly expressed cytokeratin 5 (Fig. 1b).

### KISS1R expression regulates metastasis in xenograft models

**Fig. 2:**
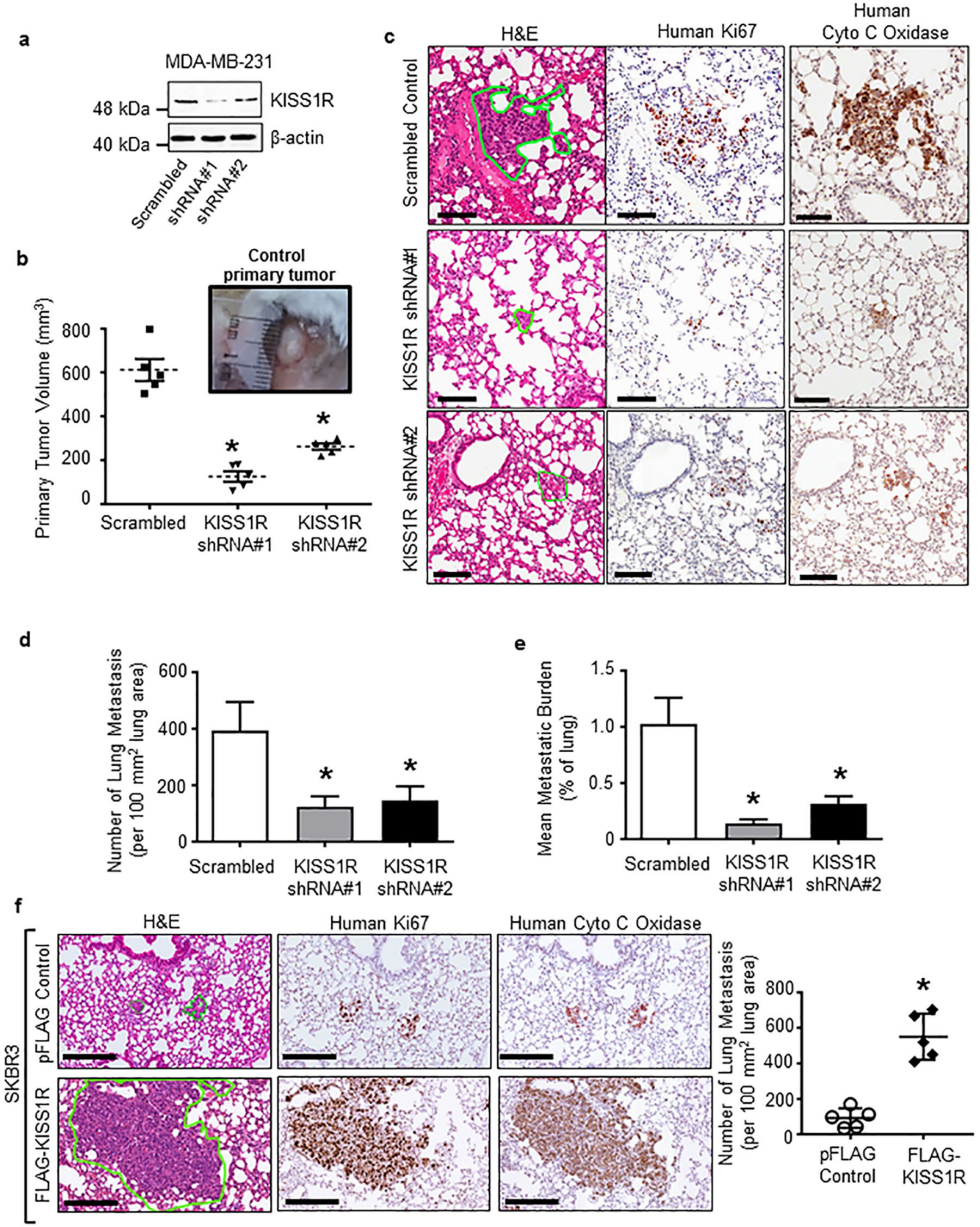
KISS1R expression regulates primary breast tumor growth and metastasis in xenograft models. **a–e** Depletion of endogenous KISS1R in human metastatic triple-negative MDA-MB-231 breast cancer cells inhibits primary tumor growth and lung metastases in a spontaneous metastasis xenograft model. **a** Representative western blot showing KISS1R expression in lysates from MDA-MB-231 cells stably expressing KISS1R shRNA and scrambled control used in xenograft experiments; β-actin, loading control. Densitometric analysis of blots shown in Supplementary Fig. 1b. **b** Primary orthotopic tumor volumes: mouse mammary fat pad, 10^6^ cells/mouse, *n* = 5 mice/group. Points represent each mouse mean tumor volume and bars represent mean volume ± SEM. **c** Representative images of lung metastasis (outlined in green, left panels) subjected to either hematoxylin and eosin, antihuman Ki67 (middle panels), or human anti-cytochrome C oxidase. Scale bar, 100 μm. The extent of lung metastasis measured by (**d**) number of lung metastases (normalized to 100 mm^2^) lung and (**e**) tumor burden in the lung (percentage of lung occupied by tumor); four sections/tissue/mouse, *n* = 5 mice per group). Bars represent lung area and number of metastases ± SEM, respectively. One-way ANOVA followed by Dunnett’s multiple comparison test: *p < 0.05. **f** KISS1R overexpression promotes lung colonization in a experimental metastasis xenograft model. Lung metastases (*n* = 5 mice/group, outlined in green) formed by human ERα-negative SKBR3 breast cancer cells stably expressing FLAG-KISS1R or pFLAG vector control. Bars represent mean ± SEM. Metastases quantified blindly in hematoxylin and eosin and human anti-cytochrome C oxidase stained slides. Student’s unpaired *t* test: *p < 0.05. Scale bars, 200 μm.

We have previously shown that KISS1R depletion using *KISS1R* shRNA in TNBC cells (MDA-MB-231 and Hs578t) reduced the expression of mesenchymal markers, cell migration, invasion, and anchorage-independent growth without affecting cell viability^19^, suggesting a role for KISS1R in malignant transformation. However, whether human KISS1R regulates metastasis is unknown. Thus, KISS1R expression was depleted in metastatic MDA-MB-231 cells (Fig. 2a, Supplementary Fig. 1c) and tested for its effect on tumor growth and metastasis in a spontaneous metastasis xenograft model using NOD/SCID/IL2 receptor γ^null^ mice^27^. Stable knockdown of KISS1R reduced primary tumor volume and the capacity of tumor cells to metastasize and colonize the lungs compared with scrambled shRNA controls (Fig. 2b–e). Lung metastases were identified using an antihuman Ki67 (a cell proliferation marker) and an antihuman cytochrome (cyto) C antibody (Fig. 2c, middle and right panels).

Since KISS1R knockdown led to reduced primary tumors (Fig. 2b), which could result in reduced metastasis, we tested whether KISS1R regulates lung colonization using an experimental metastasis xenograft model. For this assay, a *gain-of-function* approach was employed using human ERα-negative luminal SKBR3 breast cancer cells (that express low levels of endogenous KISS1R^18,27^), and stably overexpressed FLAG-KISS1R similar to levels observed in TNBC MDA-MB-231 cells^18,25,27^. Using this approach, we previously showed that KISS1R overexpression induced KP secretion and promoted an EMT-like event resulting in an upregulation of mesenchymal and cell survival markers, and increased cell invasion in vitro^18,25,27^. Following tail vein injection, mice injected with SKBR3FLAG-KISS1R cells had a significantly higher number of lung metastases compared with mice injected with controls (Fig. 2f). Taken together, these results reveal that human KISS1R induces primary tumor growth and metastasis.

### KISS1R modulates the expression of key regulators of glutamine metabolism in ERα-negative breast cancer cells

**Fig. 3:**
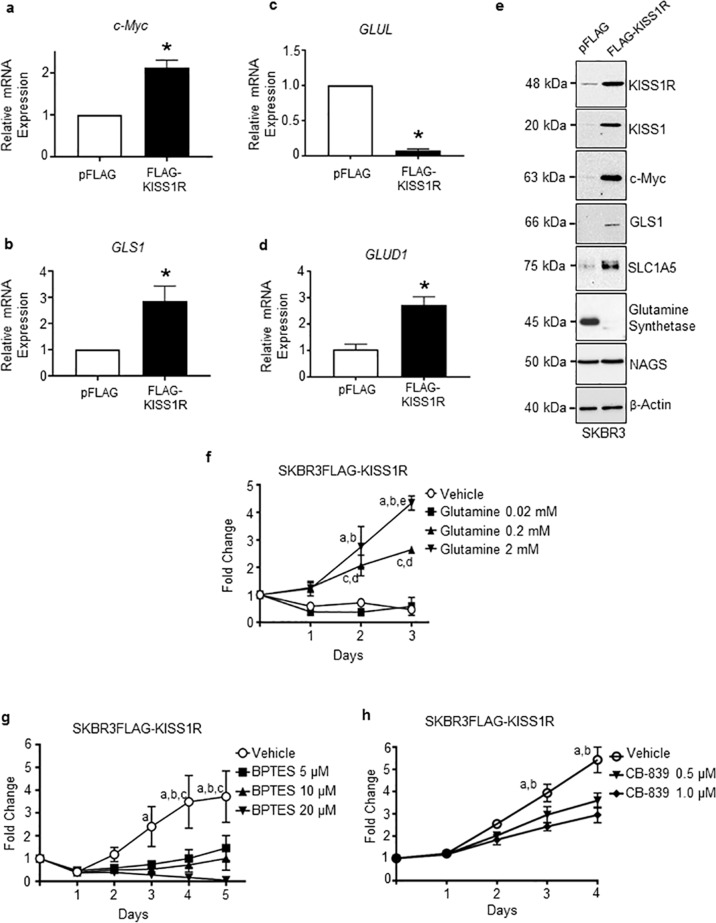
KISS1R regulates the expression of key proteins involved in glutamine metabolism. Relative mRNA expression of genes regulating glutamine metabolism by RT-qPCR in SKBR3FLAG-KISS1R breast cancer cells and SKBR3pFLAG vector controls: **a** *c-Myc*, **b** *GLS1* (encoding glutaminase), **c** *GLUL* (encoding glutamine synthetase), and **d** *GLUD1* (encoding glutamate dehydrogenase). Columns represent mean relative mRNA expression, normalized to β-actin ± SEM; Student’s unpaired *t* test, *p < 0.05. (*n* = 6). **e** Representative western blot showing the expression of KISS1R, KISS1, c-Myc, GLS1, glutamine transporter SLC1A5, and glutamine synthetase in lysates made from SKBR3 cells stably expressing FLAG-KISS1R or pFLAG vector control. See Supplementary Fig. 1c–j for quantification of blots (*n* = 4). **f** Effect of glutamine deprivation on SKBR3FLAG-KISS1R cells growth. Cells (SKBR3FLAG-KISS1R and SKBR3pFLAG controls) were grown in glutamine-free media or treated with different concentrations of glutamine and counted each day for 3 days to assess cell proliferation. Differences in cell count are expressed as fold change, calculated by dividing the number of cells from each corresponding day, by that of day 0 (*n* = 3). See Supplementary Fig. 2h for glutamine deprivation assay on SKBR3pFLAG controls. *p < 0.05; Two-way ANOVA followed by Bonferroni post hoc test: **a** glutamine 2 mM vs. 0 mM; **b** glutamine 2 mM vs. 0.02 mM; **c** glutamine 0.2 mM vs. 0 mM; **d** glutamine 0.2 mM vs. 0.02 mM; **e** glutamine 2 mM vs. 0.2 mM. Cell proliferation curves of SKBR3FLAG-KISS1R cells treated daily with glutaminase inhibitors, **g** BPTES and **h** CB-839 *p < 0.05; two-way ANOVA with multiple comparisons followed by Bonferroni post hoc test. Mean and SEM shown; (*n* = 4); **a** vehicle vs. 20 μM; **b** vehicle vs. 10 μM; **c** vehicle vs. 5 μM. See Supplementary Fig. 2i for BPTES treatment in SKBR3pFLAG controls. For **h**: **a** vehicle vs. 0.5 μM; **b** vehicle vs. 1 μM.

We then sought to determine whether KISS1R regulated the glutamine utilization to thereby support tumor growth. The major glutamine transporter expressed by TNBC tumors is the SLC1A5 (also known as ASCT2)^32^. Upon entry into the cell, glutamine is converted to glutamate by glutaminase (GLS1). Glutamate can be converted to generate α-ketoglutarate, a TCA cycle intermediate by glutamate dehydrogenase 1 (encoded by GLUD1)^2^. KISS1R overexpression in ERα-negative SKBR3 cells resulted in a significant increase in the expression of *c-Myc*, *GLS1*, *GLUD1*, but decreased expression of glutamine synthetase (encoded by *GLUL* gene) that converts glutamate to glutamine (Fig. 3a–e; Supplementary Fig. 1d–j). This suggests that KISS1R overexpressing cells lack the ability to synthesize glutamine. GLUD1 is essential for sustaining the TCA cycle in rapidly proliferating cells and is upregulated in breast cancer^37^. These data suggest that KISS1R overexpression may sustain the TCA cycle required for cell growth and proliferation. Glutamate can also be converted to a co-factor in the urea cycle, N-acetylglutamate, by N-acetylglutamate synthase (NAGS). However, NAGS expression was similar between the two cell lines (Fig. 3e, Supplementary Fig. 1j), indicating that KISS1R differentially regulates the expression of key glutamine metabolism enzymes. Conversely, KISS1R depletion in TNBC MDA-MB-231 cells revealed a reduction in the levels of c-Myc, GLS1, and SLC1A5 in KISS1R knocked-down cells versus controls (Supplementary Fig. 2a–e). Taken together, these data suggest a role for KISS1R in regulating glutamine metabolism pathway in ERα-negative breast cancer cells.

### KISS1R promotes the dependence on glutamine for growth

To determine whether KISS1R expressing cells require glutamine for growth, the effect of glutamine deprivation was examined on cell growth. SKBR3FLAG-KISS1R cells and controls were grown in glutamine-free medium and treated with increasing concentrations of glutamine daily over 72 h. Glutamine deprivation (0 and 0.02 mM glutamine) significantly inhibited SKBR3FLAG-KISS1R cell growth compared with cells grown in 0.2 or 2 mM glutamine, the latter being the glutamine concentration in cell culture media (Fig. 3f). In contrast, no changes were observed in growth of SKBR3pFLAG controls upon removal of glutamine (Supplementary Fig. 2h). Then, the effects of the GLS1 inhibitor BPTES was examined, which triggers cellular glutamate depletion^31,38^. Analysis of cell growth curves revealed that BPTES treatment (≥5 μM) significantly suppressed the growth of SKBR3FLAG-KISS1R cells in contrast to controls (Fig. 3g, Supplementary Fig. 2i). Similar observations were made with CB-839, another GLS inhibitor that is currently being tested in Phase I/II clinical trials in various cancers and shown to attenuate TNBC tumor growth in xenografts^39^. CB-839 treatment significantly inhibited cell growth (Fig. 3h). This is in agreement with reports that luminal-type cells (such as the SKBR3 cells) are relatively glutamine independent. In contrast, basal-type SKBR3FLAG-KISS1R cells exhibit glutamine-dependence for growth.

### KISS1R promotes glutamine metabolism in tumors

**Fig. 4:**
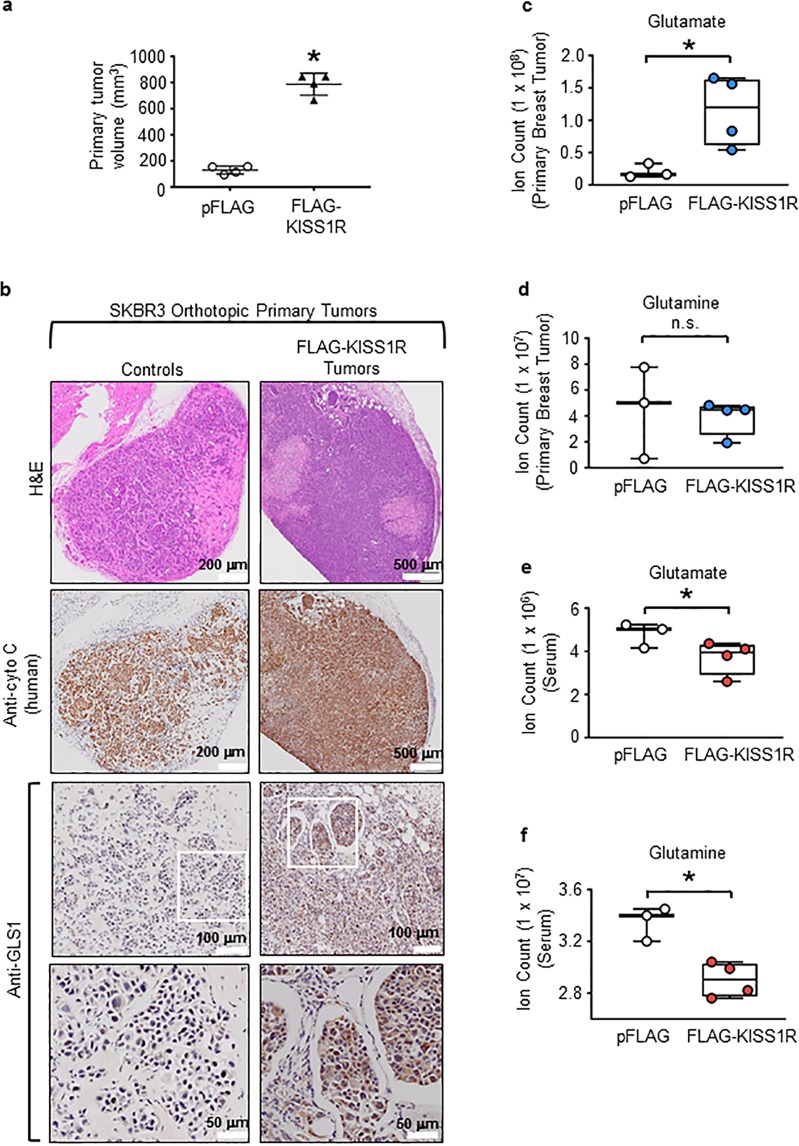
KISS1R expression promotes primary breast tumor growth and glutamine metabolism in an orthotopic xenograft model. Six-week-old immunocompromised mice were injected with SKBR3pFLAG controls (2 × 10^6^ cells/mouse) or SKBR3FLAG-KISS1R cells (10^6^ cells/mouse) into the mammary fat pad. **a** Primary tumor volume after 6 weeks for animals injected with SKBR3FLAG-KISS1R cells and 8 weeks for animals injected with SKBR3pFLAG controls. **b** Representative images of orthotopic primary tumors subjected to hematoxylin and eosin, antihuman cytochrome C oxidase, or antihuman glutaminase (GLS1); magnified section in white boxed area shown in image below. Metabolites in primary breast tumors (**c**, **d**) and serum (**e**, **f**), respectively, from xenografts: **c**, **e** glutamate and **d**, **f** glutamine levels measured by LC–MS. Student’s unpaired *t* test, *p < 0.05. (*n* = 4 mice for SKBR3FLAG-KISS1R xenografts; *n* = 3 for pFLAG control xenografts).

To investigate whether KISS1R regulates tumor metabolism, the effect of KISS1R overexpression on primary tumor metabolism was determined using an orthotopic xenograft model. Primary breast tumor volumes were significantly higher in mice injected with SKBR3FLAG-KISS1R cells compared with mice injected with controls (Fig. 4a, Supplementary Fig. 3a). Human tumor cells were identified in primary tumors (Fig. 4b). Immunohistochemical analysis revealed SKBR3FLAG-KISS1R primary tumors expressed GLS, in contrast to control tumors (Fig. 4b).

In order to uncover metabolic differences contributing to the distinct phenotypes observed between SKBR3FLAG-KISS1R primary tumors and controls, a global, untargeted metabolomic analysis of tumors was conducted. KISS1R overexpression resulted in significantly higher glutamate levels in the primary tumors compared with control tumors (Fig. 4c), whereas there was no change in glutamine levels (Fig. 4d). Both serum glutamate and glutamine levels were significantly decreased in SKBR3FLAG-KISS1R xenografts compared with controls (Fig. 4e, f). A possible explanation for this is that the primary tumor utilizes more glutamine from the serum to promote glutaminolysis, resulting in the increased glutamate content within the tumor. This might also be due to the elevated levels of glutaminase observed in SKBR3FLAG-KISS1R tumors (Figs. 3b, e and 4b). Therefore, these data suggest that KISS1R overexpression promotes glutamine metabolism in primary tumors by upregulating glutaminase expression to thereby promote tumor growth.

**Fig. 5:**
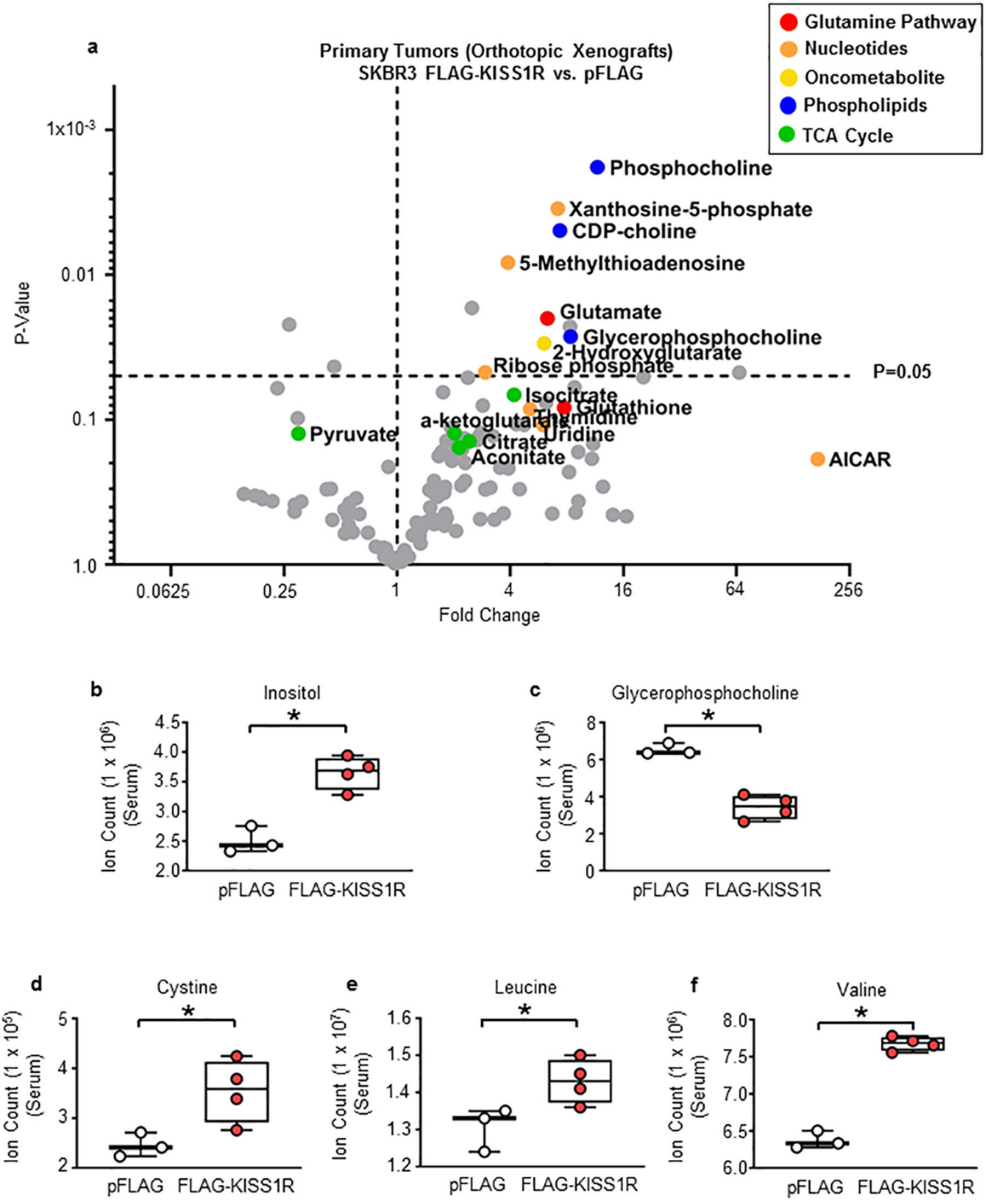
Quantitative analysis of primary tumor and serum metabolites from SKBR3FLAG-KISS1R and SKBR3pFLAG control orthotopic xenografts. **a** Volcano plot comparing metabolite levels measured by LC–MS in primary tumors of SKBR3FLAG-KISS1R xenografts versus those of SKBR3pFLAG controls. The fold change between the two groups and *p* values for each metabolite are plotted and represented in a bar graph (Supplementary Fig. 3b). Fold changes >1 and <1 indicate increases and decreases, respectively, in metabolite levels in SKBR3FLAG-KISS1R and control tumor groups. Student’s two-tailed *t* test, *p < 0.05. Metabolites in serum: **b** inositol, **c** glycerophosphocholine, **d** cysteine, **e** valine, and **f** leucine.

Metabolomic analysis of primary tumors revealed that several metabolites in the glutamine, nucleotide, and lipid synthesis pathways and TCA cycle were significantly elevated in the SKBR3FLAG-KISS1R tumors compared with control tumors (Fig. 5a). In addition, membrane phospholipids such as CDP-choline, phosphocholine, and glycerophosphocholine were significantly elevated in SKBR3FLAG-KISS1R primary tumors (Fig. 5a, Supplementary Fig. 3b). Serum level of inositol was higher, whereas glycerophosphocholine level was decreased in SKBR3FLAG-KISS1R xenografts, compared with controls, likely due to elevated uptake by tumors (Fig. 5b, c). This suggests an enhanced lipid synthesis that is characteristic of malignant tumors^40,41^. No change in glycolytic metabolites was observed in SKBR3FLAG-KISS1R tumors versus control tumors (Supplementary Fig. 3b).

In particular, two metabolites, 2-hydroxyglutarate and 5′-methylthioadenosine were identified to be significantly increased with KISS1R overexpressing primary tumors compared with controls (6.1 and 3.9-fold changes, respectively; Fig. 5a, Supplementary Fig. 3b). The oncometabolite 2-hydroxyglutarate is markedly elevated in ERα-negative human breast tumors of the basal-like/mesenchymal subtype and this has been linked to poor clinical outcome, c-Myc activation, and glutamine dependence^42^. The nucleoside 5′-methylthioadenosine can be used for purine synthesis or enter the methionine salvage pathway. Methionine is an essential amino acid required for protein synthesis, methylation of DNA, and polyamine synthesis^43^. Methionine can also be converted to the amino acid cysteine. The levels of cysteine as well as branched-chain amino acids valine and leucine were elevated in serum from SKBR3FLAG-KISS1R xenografts versus controls (Fig. 5d–f). These branched-chain amino acids can be metabolized to succinyl-CoA or acetyl-CoA and used in the TCA cycle^44^. Taken together, these results highlight KISS1R’s role in metabolic reprograming of primary tumors in vivo.

### KISS1R expression promotes glutaminolysis and TCA cycle activity in cultured ERα-negative breast cancer cells

**Fig. 6:**
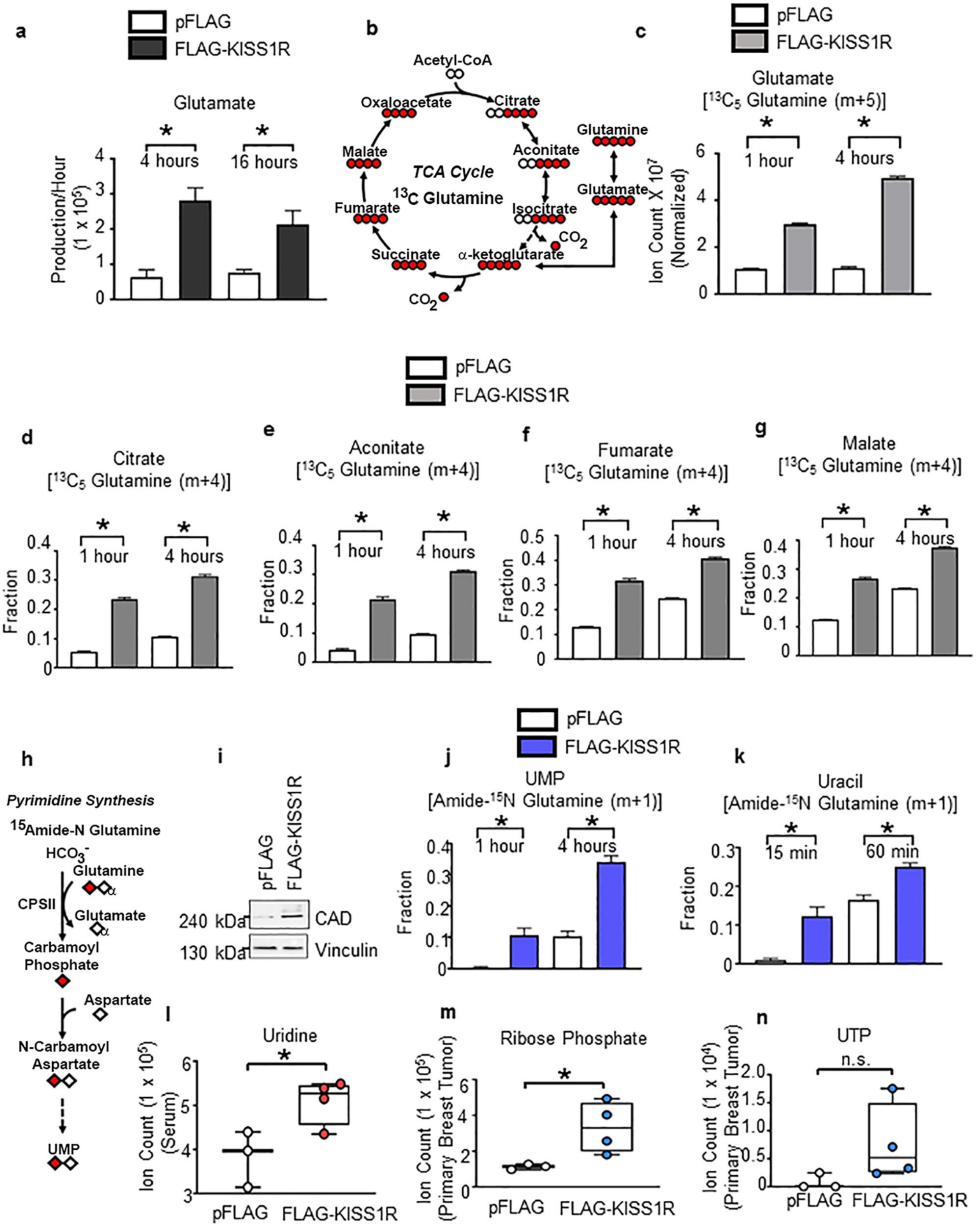
KISS1R expression promotes glutaminolysis, increases glutamine flux to tricarboxylic acid (TCA) cycle metabolites, and stimulates glutamine-dependent nucleotide synthesis. **a** Production rate of glutamate measured in the conditioned media after 4 or 16 h of culturing SKBR3pFLAG controls or SKBR3FLAG-KISS1R cells grown in serum-free media (unlabeled). **b** Schematic showing the transfer of carbon atoms of the ^13^C_5_-labeled glutamine tracer used to detect glutamine flux into TCA cycle intermediates; labeled carbon atoms (red) and unlabeled carbon atoms (white). **c** Glutamate pool size from ^13^C_5_ glutamine tracer in cells. **d–h** Relative fractions of ^13^C_5_ glutamine tracer in TCA cycle metabolites **d** citrate, **e** aconitate, **f** fumarate, and **g** malate. Normalization of pool size was done to pack cell volume per dish. Mean ± SEM shown (*n* = 3). *p < 0.05, Student’s unpaired *t* test. **h** Schematic of the pyrimidine synthesis pathway showing the contribution of the amide nitrogen using a ^15^N-glutamine tracer. Diamonds indicate nitrogen, labeled for amide (red) and unlabeled (white). Diamond with subscript α denotes the alpha nitrogen of glutamine. **i** Representative western blot of the CAD enzyme complex in lysates from SKBR3FLAG-KISS1R and SKBR3pFLAG vector control cells. See Supplementary Fig. 2f for quantification of blots. Fraction of glutamine’s amide nitrogen contributing to pyrimidine synthesis metabolites: **j** UMP and **k** uracil in cultured SKBR3FLAG-KISS1R and SKBR3pFLAG control cells (*n* = 3). Levels of pyrimidine synthesis metabolites measured by LC–MS in orthotopic breast tumors and serum from xenografts: **l** serum uridine; primary tumor (**m**), UTP (**n**) ribose phosphate. Student’s unpaired *t* test, *p < 0.05. (*n* = 4 mice for SKBR3FLAG-KISS1R xenografts; *n* = 3 for SKBR3pFLAG control xenografts).

Next, metabolic analysis was conducted using cultured SKBR3FLAG-KISS1R and control cells. In support of the in vivo data, SKBR3FLAG-KISS1R cells were found to produce more glutamate, suggesting increased glutamine metabolism (Fig. 6a). Using a ^13^C_5_-glutamine tracer, the utilization of glutamine carbon to form metabolites was traced (Fig. 6b). Glutamate pool size (calculated after normalization to the pack cell volume) was significantly larger in the SKBR3FLAG-KISS1R cells compared with controls (Fig. 6c). Glutamine can be converted to α-ketoglutarate and thereby provide four carbons for TCA metabolites. We observed greater labeling in TCA metabolites (e.g., citrate, aconitate, fumarate, and malate) in SKBR3FLAG-KISS1R cells, relative to controls (Fig. 6d–g), suggesting increased glutamine contribution to TCA cycle intermediates upon KISS1R expression.

### KISS1R expression drives glutamine-dependent nucleotide synthesis

**Fig. 7:**
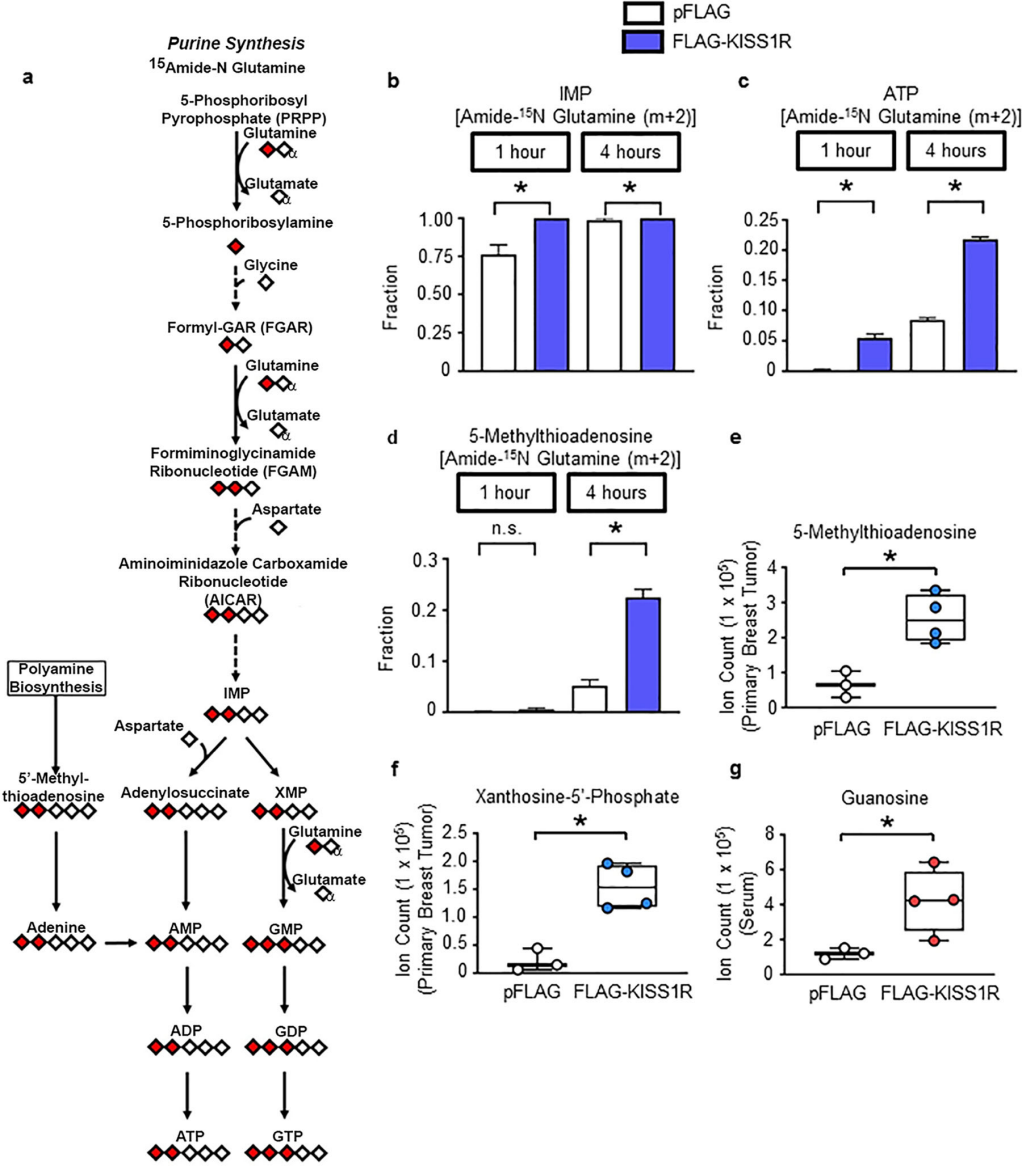
KISS1R expression promotes purine synthesis. **a** Diagram of the purine synthesis pathway, including the amide nitrogen glutamine tracer. Diamonds indicate nitrogen, labeled for amide (red) and unlabeled (white). Diamond with subscript α denotes the alpha nitrogen of glutamine. Fractional enrichment of glutamine’s amide nitrogen contributing to metabolites in purine synthesis in SKBR3FLAG-KISS1R and pFLAG control cells (*n* = 3): **b** IMP, **c** ATP, and **d** 5-methylthioadenosine. Metabolite levels in orthotopic primary tumors of **e** 5-methylthioadenosine and **f** xanthosine-5′-phosphate and **g** serum guanosine levels in SKBR3FLAG-KISS1R and SKBR3pFLAG control xenografts (*n* = 4 mice for FLAG-KISS1R xenografts; *n* = 3 for pFLAG controls). Mean ± SEM shown. *p < 0.05, Student’s unpaired *t* test.

Glutamine is an important nitrogen source in the synthesis of pyrimidines (Fig. 6h) and purines (Fig. 7a). The first rate-limiting step in de novo pyrimidine synthesis requires the enzyme carbamoyl phosphate synthase II (CPSII) that together with aspartate transcarbamylase and dihydroorotase, forms the trifunctional CAD enzyme complex. CAD protein (Fig. 6i, Supplementary Fig. 2f) and *CPSII* mRNA levels (Supplementary Fig. 2g) were significantly upregulated in SKBR3FLAG-KISS1R cells relative to controls, indicating that the pyrimidine synthesis pathway might be altered upon expression of KISS1R.

Glutamine is converted to glutamate and donates its amide (γ) nitrogen for nucleotide biosynthesis. Thus, to examine whether KISS1R regulates pyrimidine synthesis, an amide ^15^N-glutamine tracer was utilized (Fig. 6h). Results revealed that uridine monophosphate and uracil have significantly increased incorporation of the amide nitrogen from glutamine in SKBR3FLAG-KISS1R cells compared with controls (Fig. 6j, k). In line with this data, SKBR3FLAG-KISS1R primary tumors from xenografts displayed a significant increase in serum levels of the nucleoside uridine (Fig. 6l). Although we did not observe a significant change in tumor UTP levels, the level of ribose phosphate, a key intermediate in de novo nucleotide synthesis was significantly elevated in the SKBR3FLAG-KISS1R tumors (Fig. 6m, n).

To determine whether KISS1R regulates glutamine utilization in purine synthesis, an amide ^15^N-glutamine tracer was utilized (Fig. 7a). Elevated KISS1R expression resulted in a significant increase in labeled IMP and ATP after 1 and 4 h compared with controls (Fig. 7b, c). A significant increase in 5′-methylthioadenosine was also observed in KISS1R overexpressing cells (Fig. 7d), as well as in primary tumors (Figs. 5a, 7e) compared with their corresponding controls. 5′-Methylthioadenosine is generated during the biosynthesis of polyamines (Fig. 7a) and generates purines. Interestingly, deprivation of 5′-methylthioadenosine inhibits TNBC metastasis^45^. Other metabolites implicated in purine synthesis, such as the intermediate xanthosine-5′-phosphate (XMP; Fig. 7f) and the nucleoside guanosine (Fig. 7g) were elevated in SKBR3FLAG-KISS1R primary tumors and serum levels, respectively. Taken together, these data suggest that KISS1R signaling reprograms metabolism in primary tumors, driving the use of the amide nitrogen from glutamine for nucleotide synthesis.

### KISS1R induces c-Myc expression to regulate cell motility and glutamate production via MAPK

**Fig. 8:**
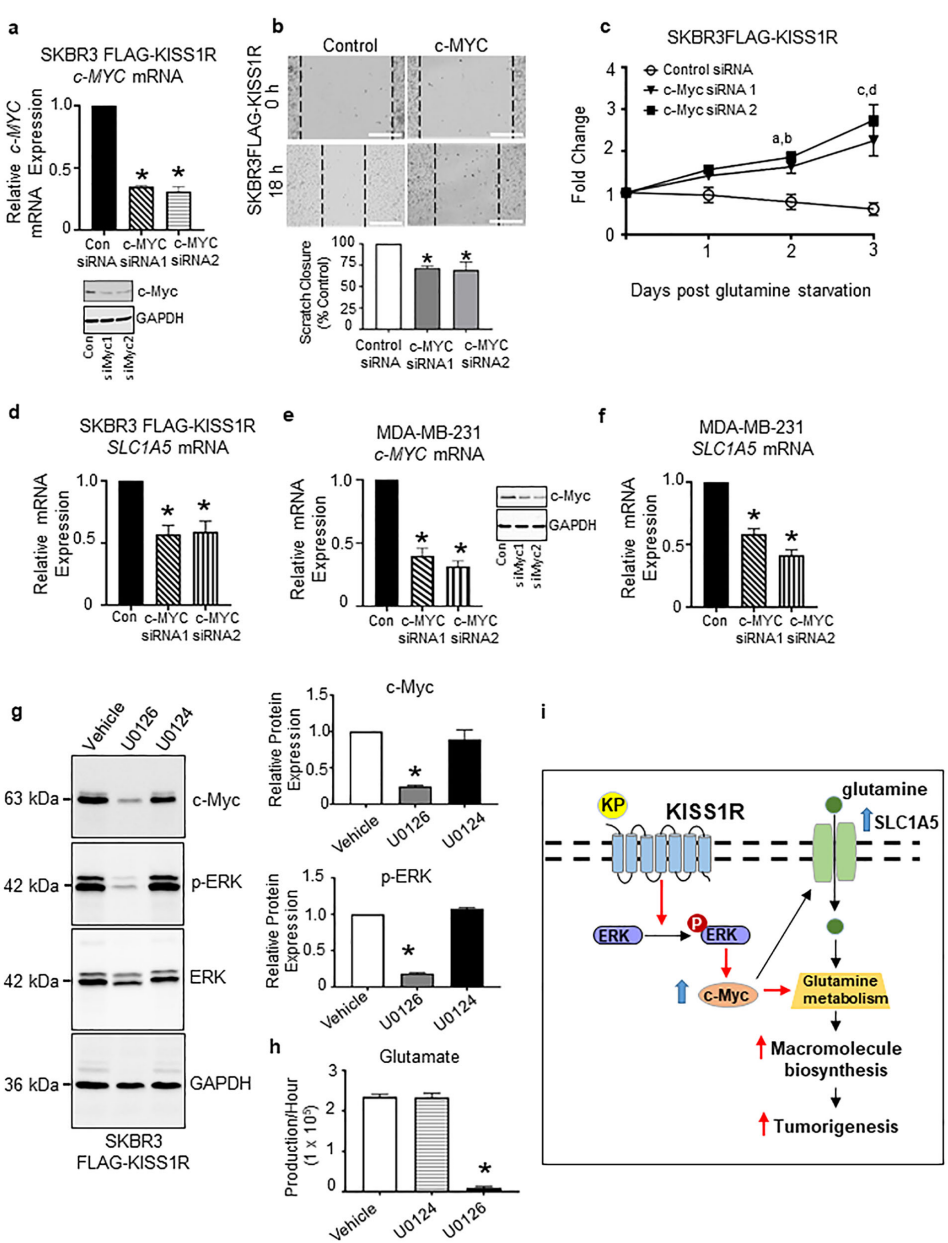
KISS1R induces c-Myc expression to regulate glutamate production via MAPK. **a** *c-MYC* mRNA and protein expression in SKBR3FLAG-KISS1R cells expressing c-MYC siRNA or control siRNA (Con) constructs, 72 h after transfection. (*n* = 5). **b** Representative images of the scratch assay to assess cell motility of SKBR3FLAG-KISS1R cells transfected with c-MYC siRNA or control siRNA at 0 and 18 h (*n* = 3). Scale bars, 300 μm. Scrambled cells migrated 548.2 ± 14.72 μM. Bars show mean ± SEM. Student’s unpaired *t* test, *p < 0.05. c-Myc-suppressed SKBR3FLAG-KISS1R cells are resistant to glutamine starvation. Cells expressing c-MYC siRNA were plated in the presence of glutamine and then cultured in the absence of glutamine and counted each day to assess cell proliferation. Differences in cell count are expressed as fold change, calculated by dividing the number of cells from each corresponding day, by that of day 0 *p < 0.05; two-way ANOVA with multiple comparisons followed by Bonferroni post hoc test. Mean and SEM shown (*n* = 3). **a**, **c** control vs. siMYC1; **b**, **d** control vs. siMYC2. **d** *SLC1A5* mRNA expression in SKBR3FLAG-KISS1R cells expressing c-MYC siRNA or control siRNA. (*n* = 5), **e** *c-MYC* mRNA and protein expression, and **f** *SLC1A5* mRNA in MDA-MB-231 cells expressing c-MYC siRNA or control siRNA constructs, 72 h after transfection. (*n* = 5). **g** Representative western blots showing endogenous c-Myc expression in SKBR3FLAG-KISS1R cells treated with vehicle, 10 µM U0126 (ERK1 and ERK2 inhibitor), and 10 µM U0124 (inactive U0126 analog). Densitometric analysis of blots shown for c-Myc normalized to housekeeping (GAPDH) and p-ERK normalized to total ERK expression (*n* = 3). **h** Glutamate production rate measured in the unlabeled conditioned media of SKBR3FLAG-KISS1R cells grown in the presence of U0126, U0124 or vehicle (*n* = 3). **i** Schematic of the KISS1R signaling pathway in cancer metabolism. Pathway shown in red is a potential mechanism by which KISS1R promotes glutamine uptake by the tumors to thereby regulate tumorigenesis.

We have previously shown that KISS1R expression in SKBR3 cells stimulates cell migration and scratch closure within 24 h, and this is not due to increased cell proliferation^25^. Since c-Myc can regulate breast tumor migration^46^, we determined the effect of c-Myc depletion in SKBR3FLAG-KISS1R cell migration. Expression of c-Myc siRNA (Fig. 8a) demonstrated a modest but significant decrease in cell migration compared with scrambled controls in a scratch assay (Fig. 8b). c-Myc has been shown to promote glutamine addiction in various cell types^47,48^. To investigate the role of c-Myc in inducing glutamine dependency of SKBR3FLAG-KISS1R cells, cells expressing c-Myc siRNA were grown in glutamine-depleted media. Knockdown of c-Myc significantly increased resistance of SKBR3FLAG-KISS1R to glutamine starvation compared with controls (Fig. 8c). We also found that c-Myc depletion in SKBR3FLAG-KISS1R and MDA-MB-231 cell lines (Fig. 8a, e) significantly reduced the expression of glutamine transporter SLC1A5 (Fig. 8d, f), although there was no change in GLS expression (Supplementary Fig. 4a, b). This suggests c-Myc appears to regulate transcription of genes involved in glutamine transport, as previously reported^48^.

It is well known that KISS1R signaling activates MAPK^10,17,25,49^ and in TNBC, KISS1R signaling stimulates the phosphorylation of ERK1/2 to promote cell invasion^19,20,27^. KISS1R overexpression activates ERK1/2 in SKBR3 cells^25^ and ERK1/2 activation stabilizes transcription factors such as c-Myc^50^. Since c-Myc protein was upregulated in SKBR3FLAG-KISS1R cells (Fig. 3e, Supplementary Fig. 1f), we determined whether KISS1R influenced c-Myc expression post transcriptionally via ERK1/2. Findings revealed that treating SKBR3FLAG-KISS1R cells with an ERK1/2 inhibitor, U0126, resulted in an inhibition of ERK activity and c-Myc expression and a concomitant decrease in glutamate production, compared with cells treated with the inactive analog U0124 or vehicle controls (Fig. 8g, h). This implies that in addition to stimulating *c-Myc* transcription (Fig. 3a), KISS1R requires MAPK signaling to induce c-Myc protein expression to thereby maintain the glutaminolytic phenotype, and addiction to glutamine as a bioenergetic substrate. Taken together, data suggest that KISS1R exerts its oncogenic functions by inducing c-Myc expression to thereby enhance glutamine metabolism and facilitate tumorigenesis (Fig. 8i).

## Discussion

The findings reported here demonstrate for the first time that human KISS1R promotes TNBC tumor growth and metastasis using preclinical xenograft models. In support of our findings, murine *Kiss1r* promotes metastasis in a mouse mammary tumor virus model^51^. KISS1R is a strong candidate for targeted therapy since it is highly expressed in patient TNBC tumors, in contrast to normal breast^25^. Importantly although infertile, mice lacking KISS1R exhibit no other overt developmental defects^10^. KISS1R is a key regulator of pubertal onset and fertility^9^ and plasma KPs in humans increase dramatically in puberty (~50–60pmol/L) and during pregnancy (~11,000 fmol/mL in the third trimester)^9,10^, where the placenta is suggested to be a major source of the circulating KPs (reviewed^10^). Despite the small cohort size, we found that plasma KP levels were elevated in TNBC. Thus, a rise in plasma KPs may influence metabolic reprogramming during oncogenic transformation, although this is yet to be tested. This suggests that KISS1R could potentially serve not only as an adjunct therapeutic target but also as a marker for surveillance of disease recurrence or metastasis, which requires further investigation. KISS1/KISS1R expression are upregulated in TNBC patient tumor biopsies and high KISS1 expression has been shown to correlate with increase in lymph node metastasis^52^. Our future studies will examine the clinical relevance of KP/KISS1R signaling in other breast cancer subtypes, which have a distinct molecular characteristics and clinical regiments, compared with TNBC^1,53^.

Previous work has shown that KISS1 and KISS1R levels are upregulated in invasive ERα-negative breast cancer cells (e.g., MDA-MB-231, Hs578T, SKBR3FLAG-KISS1R) in contrast to weakly invasive, ERα-positive luminal breast cancer cells (T47D, MCF7) or ERα-negative luminal SKBR3 cells^25^. KP failed to stimulate invasiveness of T47D or MCF7 cells, or upon reexpression of ERα in TNBC MDA-MB-231 cells^18^; in the latter, estradiol resulted in a downregulation of KISS1/KISS1R levels^18,21^. This negative regulation by estradiol signaling also occurs in the brain^54^. In line with these observations, placental KPs induce ERα-negative breast cancer cell invasion, whereas cell invasion of ERα-positive breast cancer cells was inhibited^55^. Interestingly, a recent study showed that TGFβ promotes TNBC cell invasion by inducing *KISS1* expression, by regulating MMP-9 levels^52^.

Another new finding is that in addition to regulating tumor growth and metastasis in vivo, KISS1R regulates metabolic changes in tumors. Compared with luminal cells e.g., ERα-negative SKBR3, basal-like TNBC cells such as MDA-MB-231 have been shown to preferentially consume more glutamine than glucose, display deregulated glutaminolysis, and require GLS for growth and survival^31,56^. GLS1 knockdown in MDA-MB-231 cells inhibits tumor growth in xenografts^31^. Our data suggest that KISS1R overexpression changes the metabolic profile of the SKBR3 cells appearing to make them more basal like and dependent on glutamine for survival. Metabolomic analyses of human primary tumor biopsies revealed elevated levels of glutamate in TNBC tumors versus ERα-positive tumors^5–7^.

The MYC oncogene is overexpressed in TNBC compared with other breast cancer subtypes^57,58^ where it promotes EMT and metastasis^46^. c-Myc is a master regulator of metabolic pathways and stimulates glutaminolysis, leading to glutamine addiction^59^ by regulating the expression of GLS^3^ and SLC1A5^32,60^. SLC1A5 is highly expressed in TNBC patients^32^ and SLC1A5 activity is critical for TNBC cell growth but not for the growth of luminal breast cancer^32^. We found that KISS1R induced the expression of c-Myc to thereby promote the glutamine-dependency phenotype. Furthermore, KISS1R appears to promote glutamine uptake by the tumors by inducing SLC1A5 transcript levels in a c-Myc-dependent manner. Glutamine is converted to glutamate by GLS1 and is used to support crucial pathways including nucleotide biosynthesis, TCA cycle, and amino acid synthesis in proliferative ERα-negative tumors (Fig. 8i). Although KISS1R-induced GLS transcription, this appears to occur in a c-Myc-independent manner. Several other studies have shown that altering c-Myc levels has no effect on GLS1 expression, similar to our findings^3,61–63^ suggesting that GLS may be regulated by KISS1R via alternate mechanisms. Our future work will evaluate the role of c-Myc in stimulating KISS1R-induced tumor growth and metastasis in vivo.

Increased KISS1R expression in primary tumor xenografts resulted in elevated levels of the oncometabolite 2-hydroxyglutarate that accumulates at higher levels in basal-like TNBC tumors versus ERα-positive tumors, is associates with poor patient prognosis^5,42^ and triggers cell transformation and epigenetic reprogramming. 2-Hydroxyglutarate accumulation induces c-Myc activation and higher glutamine utilization via GLS1^60^. 2-Hydroxyglutarate can also activate mammalian target of rapamycin complex 1 (mTORC1)^64^. In doing so, mTORC1 promotes glutamine anaplerosis, replenishing the TCA cycle metabolites by activating GLUD for the production of α-ketoglutarate from glutamate^65^. In addition, 2-hydroxyglutarate can increase reductive carboxylation to generate citrate and supports the synthesis of acetyl-CoA and lipids^2^. This might be a possible explanation for the increased membrane lipids observed in primary SKBR3FLAG-KISS1R tumor xenografts. Although KISS1R expression modulates *GLUD* transcript levels, it remains to be investigated whether KISS1R regulates mTORC1.

In summary, this study highlights the role of KISS1R as a driving force in TNBC metastasis, promoting the glutamine dependence of tumors to support their growth. Compared with other breast cancer subtypes, TNBC patients have the worst prognosis with no approved targeted therapies^1^. Inhibitors for GLS as well as for the glutamine transporter SCL1A5 are currently in clinical trials for the treatment of TNBC patients^66–68^. Thus, a combined strategy that targets KISS1R signaling coupled to the use of inhibitors of glutamine metabolism are vital to minimize toxicity and may hold great therapeutic promise to improve the clinical management of TNBC patients.

## Supplementary information

Supplemental Figure Legends

Supplementary Figure 1

Supplementary Figure 2

Supplementary Figure 3

Supplementary Figure 4
