# Supplementary material for: G protein-coupled kisspeptin receptor induces metabolic reprograming and tumorigenesis in estrogen receptor-negative breast cancer
Source: Cell Death Dis. 2020 Feb 7;11(2):106. doi: 10.1038/s41419-020-2305-7 (PMC7005685; doi:10.1038/s41419-020-2305-7)
Supplement: Supplementary file 1 — Supplemental Figure Legends [file 41419_2020_2305_MOESM1_ESM.docx]

**Dragan et al. CDDIS-19-2607RR**

**Supplemental Figure Legends**

**Supplementary Figure 1. Expression of key regulators of glutamine metabolism in human TNBC primary tumor biopsies and breast cancer cells. (a)** Representative Western blots showing the expression of endogenous c-Myc and GLS1 in human TNBC primary tumor biopsies and in MDA-MB-231 cell lysates (positive control). Total of 12 TNBC tumors were used for analysis (see Fig 1a; data from 6 samples shown here). **(b)** Representative Western blot showing the expression of endogenous cytokeratin 18 (epithelial marker) in healthy human breast biopsies (n=6). **(c)** Densitometric analysis of blots shown in Fig. 2a of human TNBC MDA-MB-231 cells stably expressing scrambled control or KISS1R shRNA (n=3). **(d-j)** Densitometric analysis of blots shown in Fig. 3e showing the expression of regulators of glutamine metabolism in human ERα-negative SKBR3 breast cancer cells expressing pFLAG controls and FLAG-KISS1R (n=4): **(d)** KISS1R, **(e)** KISS1, **(f)** c-Myc, **(g)** glutaminase, **(h)** SLC1A5 **(i)** glutamine synthetase and **(j)**NAGS. Mean ± SEM shown, Student’s unpaired t-test, *p < 0.05.

**Supplementary Figure 2. Regulators of glutamine metabolism in human metastatic TNBC cells depleted of KISS1R. (a)** Representative blots showing the endogenous expression of proteins in lysates from MDA-MB-231 cells stably expressing KISS1R shRNA and scrambled control. **(b-e)** Densitometric analysis of blots on left showing the expression of **(b)** c-Myc, **(c)** glutaminase (GLS1), **(d)** SLC1A5, and **(e)** NAGS (n=4)**;** mean ± SEM shown, Student’s unpaired t-test *p < 0.05. **(f)** Quantification of blots from Fig 6i (n=4). (**g**) Relative mRNA expression of *CPSII* by RT-qPCR in SKBR3FLAG-KISS1R cells and SKBR3pFLAG controls (n=4). Mean ± SEM shown. *p < 0.05, Student’s unpaired t-test. (**h**) Effect of glutamine deprivation on growth curves of SKBR3pFLAG controls; cells were grown in glutamine-free media or with 2 mM glutamine for 3 days. Fold change was calculated by dividing cell numbers of days 1, 2, and 3 by day 0 for each condition; mean ± SEM shown (n=3). *p < 0.05; two-way ANOVA with multiple comparisons followed by Bonferroni post-hoc test. **(i)** Growth curves of SKBR3pFLAG controls treated daily with vehicle or BPTES at 5 μM over 5 days. Fold change was calculated by dividing cell numbers of days 1 to 5 by that of day 0 for each condition (n=3). *p < 0.05; two-way ANOVA with multiple comparisons followed by Bonferroni post-hoc test.

**Supplementary Figure 3. Growth and metabolic profiling of primary tumor xenografts. (a)** Primary tumor growth in pFLAG and SKBR3FLAG-KISS1R orthotopic xenografts. Primary tumor volume of animals injected with SKBR3FLAG-KISS1R cells or controls. **(b)** Bar graph showing metabolites measured by LC–MS in primary tumors from mice bearing SKBR3FLAG-KISS1R tumors vs. SKBR3pFLAG control tumors. Relative fold change of metabolites (expressed as a ratio) between the two groups is shown along the y-axis. Significant changes are indicated by an asterisk preceding the metabolite name (n=4 mice for SKBR3FLAG-KISS1R xenografts; n=3 for pFLAG control xenografts). Student’s unpaired t-test, *p < 0.05.

**Supplementary Figure 4. Effect of c-Myc knock-down on GLS expression.** *GLS* mRNA expression in **(a)** SKBR3FLAG-KISS1R cells or **(b)** MDA-MB-231 cells expressing c-MYC siRNA or control siRNA cells, 72h after transfection (n=5).
