# Supplementary figures and images for: G protein-coupled kisspeptin receptor induces metabolic reprograming and tumorigenesis in estrogen receptor-negative breast cancer
Source: Cell Death Dis. 2020 Feb 7;11(2):106. doi: 10.1038/s41419-020-2305-7 (PMC7005685; doi:10.1038/s41419-020-2305-7)

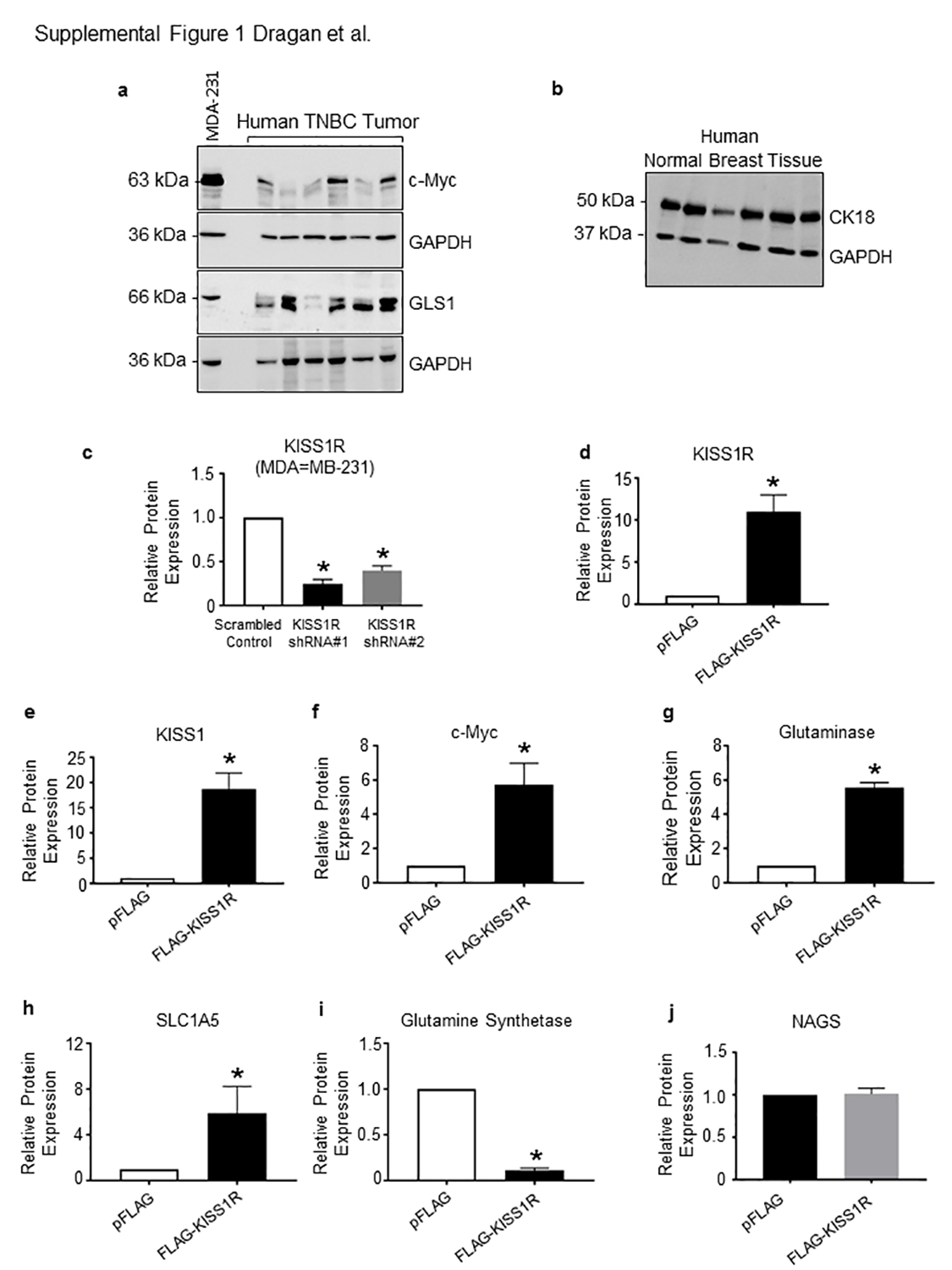

Supplement: Supplementary file 2 — Supplementary Figure 1 [file 41419_2020_2305_MOESM2_ESM.tif]

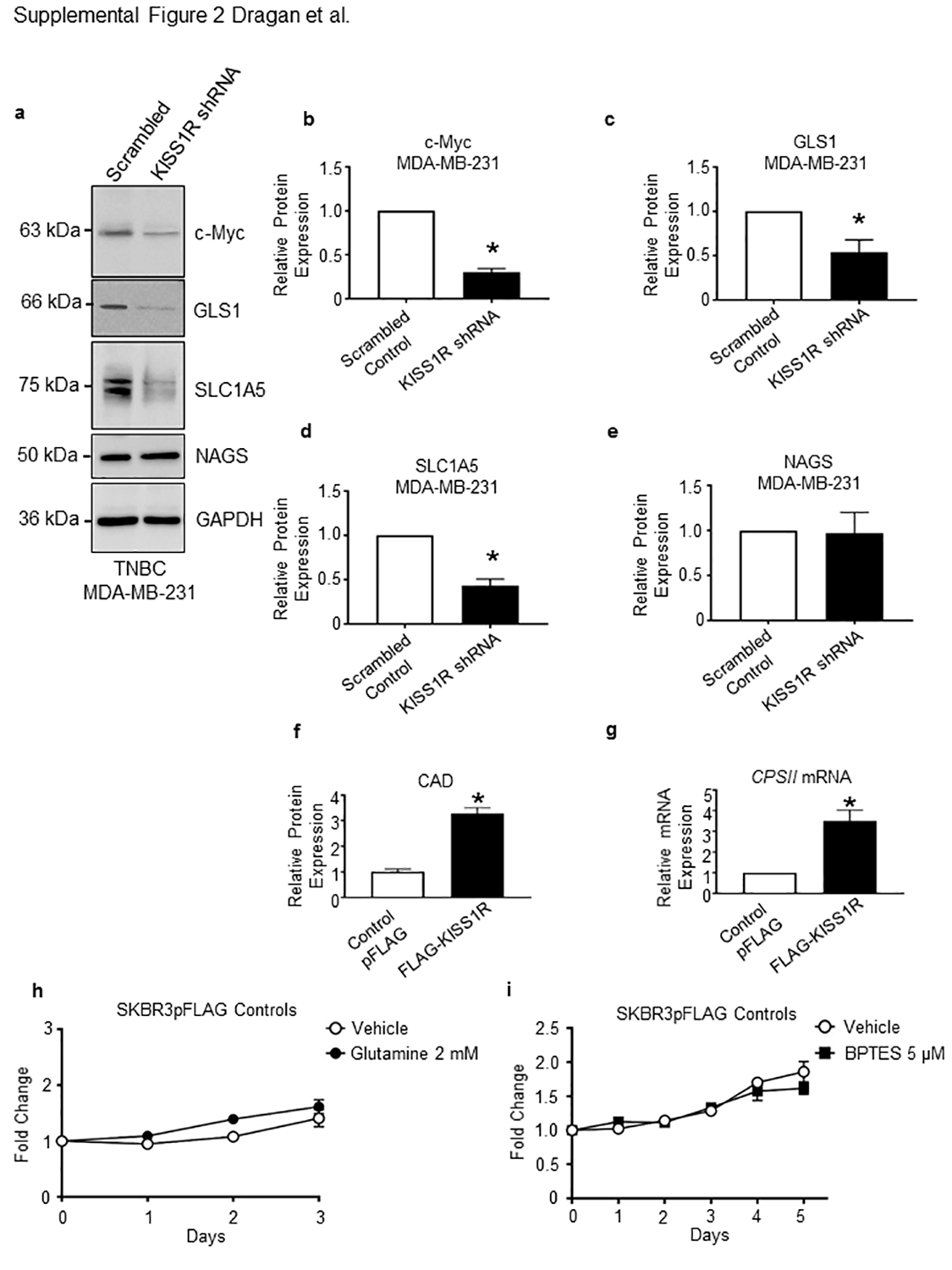

Supplement: Supplementary file 3 — Supplementary Figure 2 [file 41419_2020_2305_MOESM3_ESM.tif]

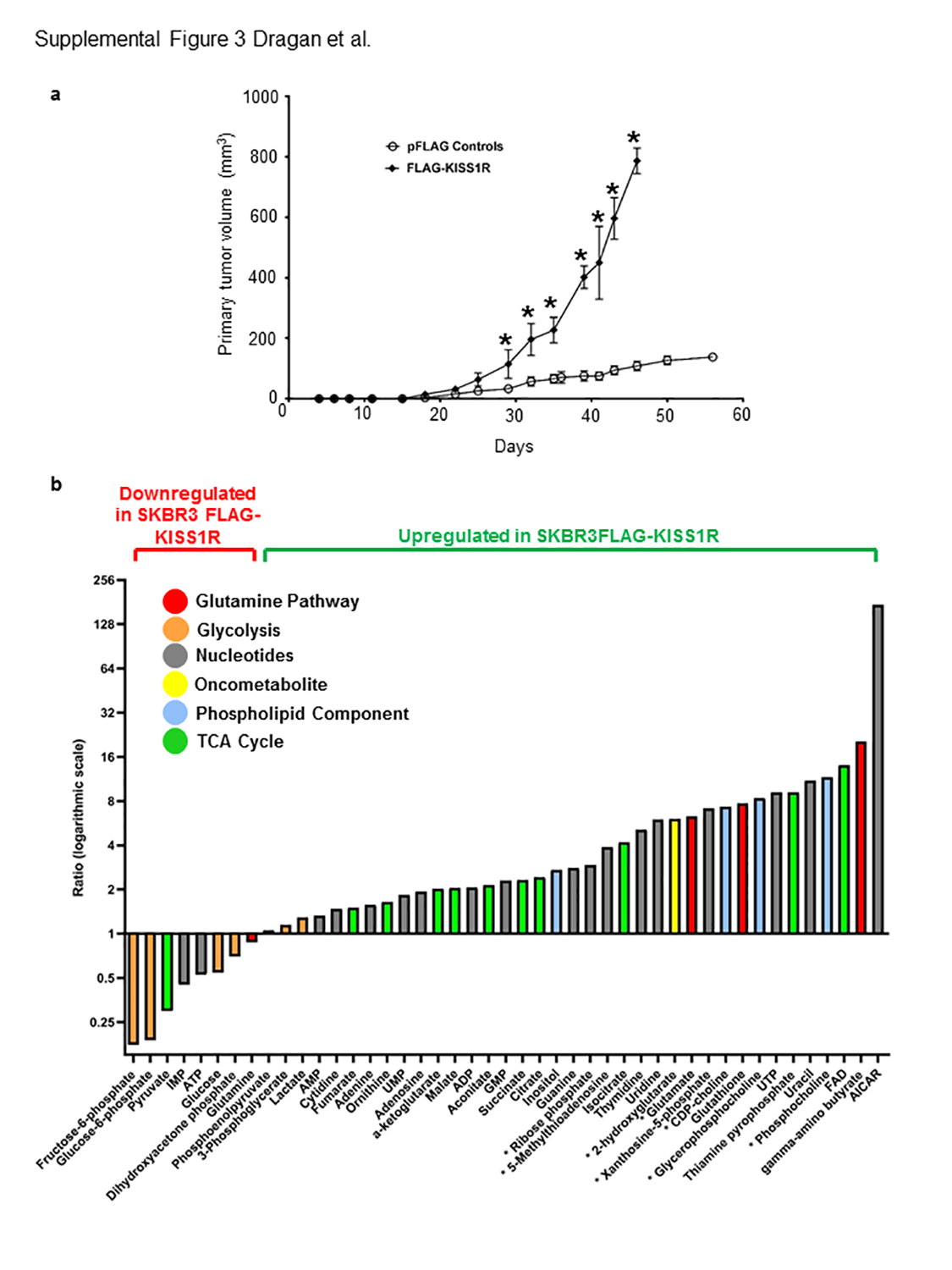

Supplement: Supplementary file 4 — Supplementary Figure 3 [file 41419_2020_2305_MOESM4_ESM.tif]

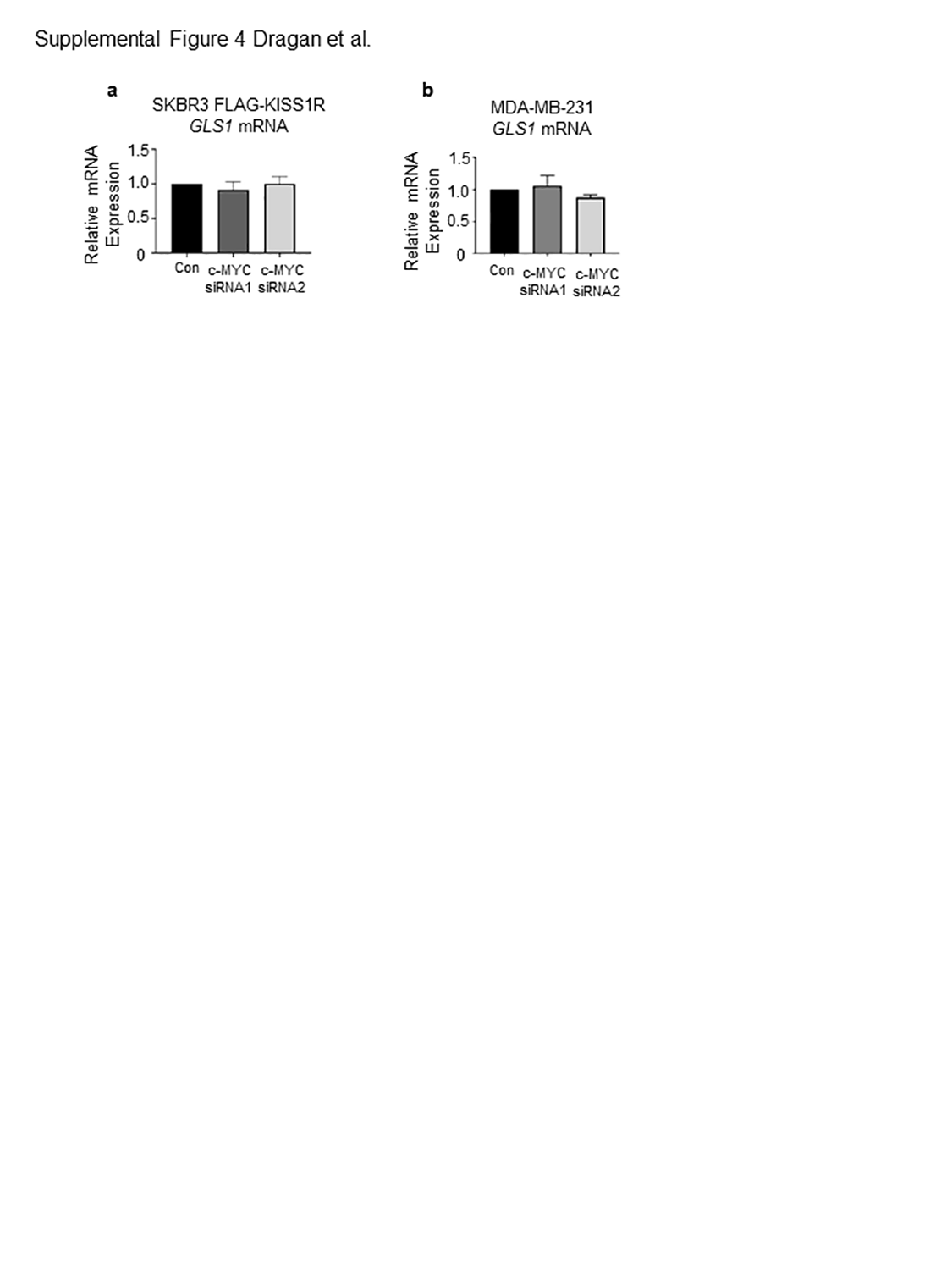

Supplement: Supplementary file 5 — Supplementary Figure 4 [file 41419_2020_2305_MOESM5_ESM.tif]
